# Mapping of ventricular tachycardia in patients with ischemic cardiomyopathy: Current approaches and future perspectives

**DOI:** 10.1002/clc.23245

**Published:** 2019-08-14

**Authors:** Claudio Pandozi, Carlo Lavalle, Maurizio Russo, Marco Galeazzi, Sabina Ficili, Maurizio Malacrida, Carlos Centurion Aznaran, Furio Colivicchi

**Affiliations:** ^1^ Division of Cardiology San Filippo Neri Hospital Rome Italy; ^2^ Boston Scientific Italy Milan Italy

**Keywords:** ablation, functional re‐entry, high‐density mapping system, ischemic cardiomyopathy, ventricular tachycardia

## Abstract

Despite the technical improvements made in recent years, the overall long‐term success rate of ventricular tachycardia (VT) ablation in patients with ischemic cardiomyopathy remains disappointing. This unsatisfactory situation has persisted even though several approaches to VT substrate ablation allow mapping and ablation of noninducible/nontolerated arrhythmias. The current substrate mapping methods present some shortcomings regarding the accurate definition of the true scar, the modality of detection in sinus rhythm of abnormal electrograms that identify sites of critical channels during VT and the possibility to determine the boundaries of functional re‐entrant circuits during sinus or paced rhythms. In this review, we focus on current and proposed ablation strategies for VT to provide an overview of the potential/real application (and results) of several ablation approaches and future perspectives.

## 1 INTRODUCTION

Radiofrequency catheter ablation is an effective treatment for drug‐refractory ventricular tachycardias (VTs) in patients with ischemic cardiomyopathy.^1^ However, although the use of three‐dimensional (3D) mapping systems and open irrigated catheters and the advent of percutaneous epicardial ablation have improved the overall success rates of these procedures, the recurrence rate of the arrhythmia remains high.^2^ In structural heart disease, VT is usually due to scar‐related re‐entry. The two commonly adopted treatment strategies are ablation during VT and substrate‐based ablation. If a re‐entrant VT is reproducible and hemodynamically tolerated, activation and/or entrainment mapping can be used to identify a critical re‐entry isthmus and ablate the VT. If VTs are not inducible or not hemodynamically tolerated, ablation can be guided by identifying the VT substrate during sinus rhythm or a paced rhythm.^3^ Substrate‐based approaches involve identifying low‐voltage areas and abnormal electrograms that represent surviving myocytes capable of supporting re‐entrant VT circuits.^4^ Comparing if substrate‐based ablation strategy to one guided predominantly by activation/entrainment mapping of inducible and hemodynamically tolerated VTs the results have been variable.^5^ However, recent studies seem to suggest that substrate‐based ablation is superior to the ablation of clinical hemodynamically stable VT, even in patients with only tolerated (clinical and/or induced) VTs.^6^, ^7^, ^8^

Mainly because of the unsuitability of a prespecified single approach in all patients and the specificity of each individual case, the problem of identifying the best approach a priori remains unsolved and a combination of the two approaches is commonly employed during VT ablation.

The aims of the present review are the following:

1. to focus on the currently proposed substrate ablation strategies for scar‐related VT in ischemic heart disease.
2. to discuss the limitation of the current substrate mapping techniques in scar definition, in abnormal electrograms recording and in the identification, in sinus rhythm, of the sites where functional circuits develops during VT:
3. to propose new strategies to curtail the limitation of current substrate mapping and ablation procedures.

## 2 ACTIVATION/ENTRAINMENT MAPPING AND SUBSTRATE MAPPING

Conventional mapping techniques (activation mapping and entrainment mapping) have been used to define the mechanism of tolerated arrhythmias and to identify a critical re‐entry isthmus and the potential ablation sites.^9^ Entrainment mapping is a technique that uses pacing to confirm the re‐entrant mechanism of VT and identify critical and noncritical areas of a VT circuit helping classify pacing sites as isthmus, bystander, and inner or outer loop sites of the VT circuit.^10^

**Figure 1:**
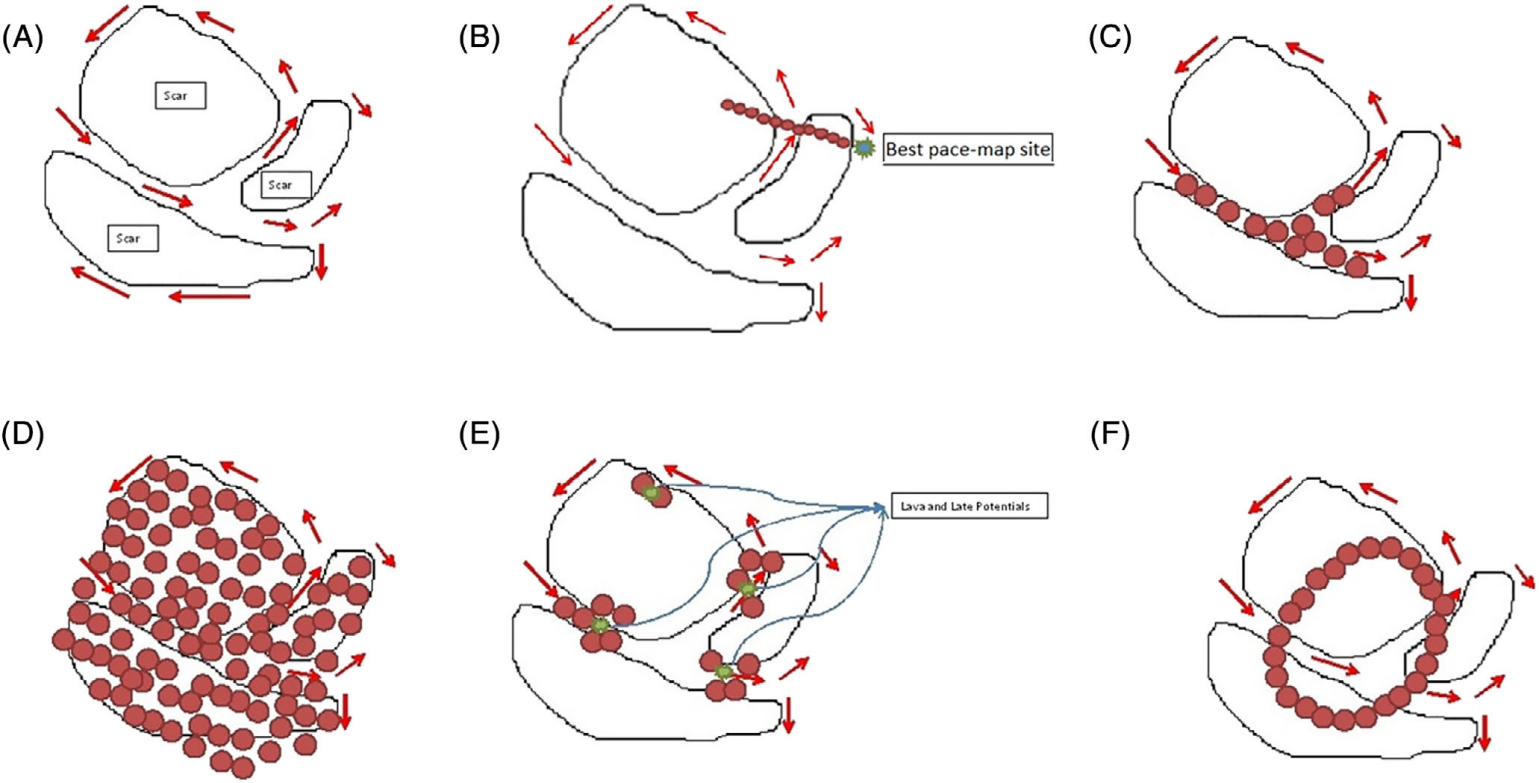
The most common strategies of ventricular tachycardia (VT) ablation. A, Schematic representation of a VT substrate. Areas of dense scar containing channels of surviving fibers forming possible VT isthmuses of re‐entrant VT circuits. B, Linear ablation lesions extended perpendicular from the border zone to the area of dense scar. C, Scar de‐channeling. D, Scar homogenization. E, Ablation of local abnormal ventricular activity (LAVA) and late potentials (LPs). F, Core isolation

Alternative ablation methods involving mapping and ablation in sinus or paced rhythm have been developed for patients with unmappable VT although they are also commonly used also in patients with tolerated VTs. Substrate‐based ablation strategies include linear lesions along the infarct border, ablation of late potentials (LPs) and local abnormal ventricular activity (LAVA), scar homogenization, scar de‐channeling, pace mapping, and scar/core isolation (Figure 1).

Linear ablation (Figure 1B) was developed in order to replicate the surgical experience of subendocardial resection.^11^ Marchlinski et al.^4^ first described the use of linear ablation lesions crossing the border zone and intersecting the best pace‐map site to target multiple unmappable VTs.

**Figure 2:**
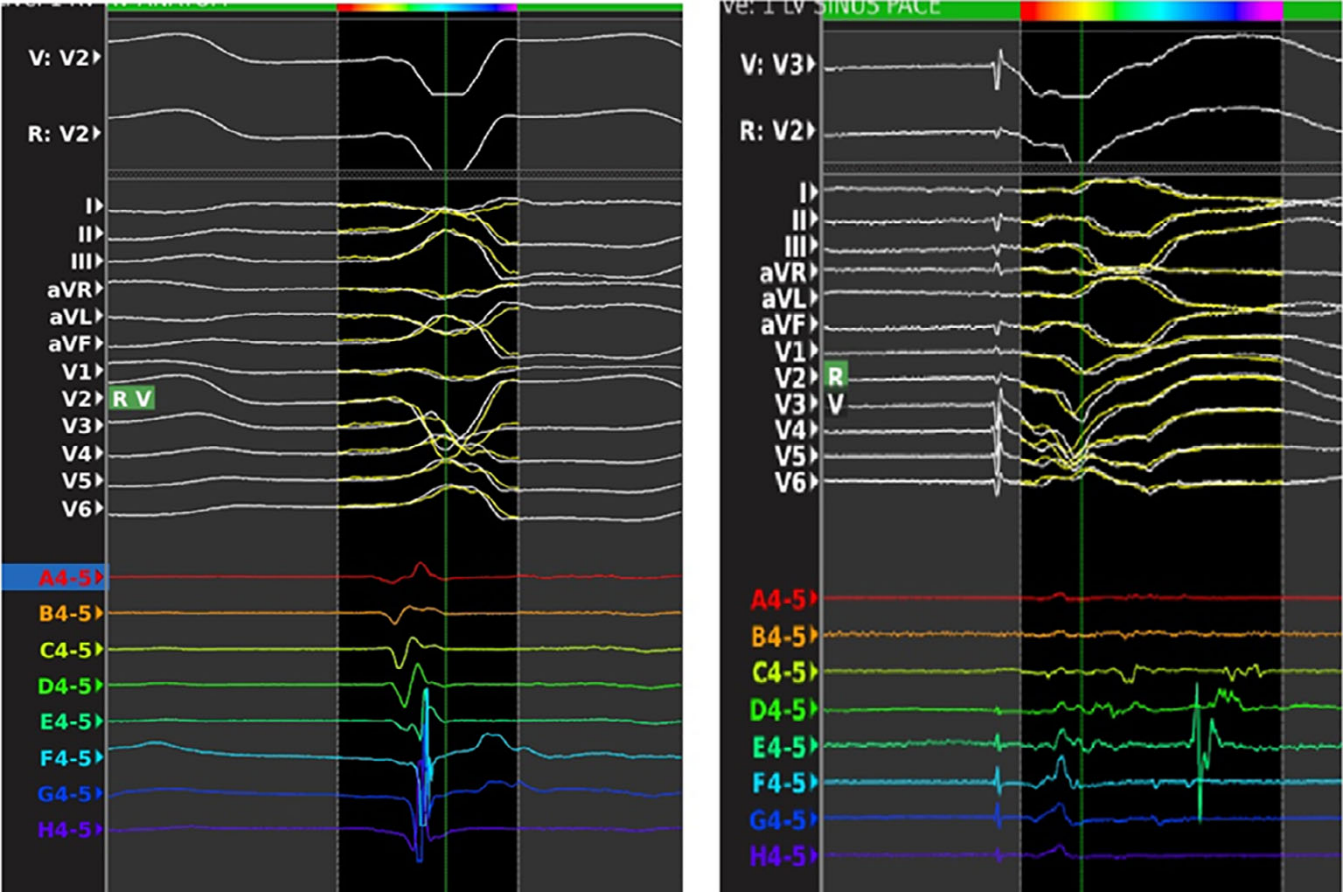
Morphology and time of onset of electrograms are related to the direction of the propagating impulse. A masked electrogram during sinus rhythm (left) becomes an evident abnormal and late potential during pacing from the right ventricular apex (right)

Ablation of LPs (Figure 1C) relates to elimination of any abnormal, fractionated electrogram with a duration that extended beyond the end of the surface QRS (Figure S1), according to the definition of Cassidy et al.^12^ modified with subsequent experiences^8^ (Figure 2A).

Ablation of local abnormal ventricular activities (Figure 1C) refers to elimination of LAVA. LAVA is a global term that incorporates all abnormal ventricular signals that represent near‐field signals of slowly conducting tissue and hence potential VT isthmuses.^13^ and occurring anytime during or after the far‐field ventricular electrogram in sinus rhythm^7^ (Figure 2B).

In the scar homogenization approach (Figure 1D), ablation lesions are empirically extended throughout the entire scar based on the substrate map defined by 3D mapping and targeting any abnormal potentials (delayed and fragmented) in normal sinus rhythm.^6^

More recently, Tung et al.^14^ and Berruezo et al.^15^ proposed a “scar de‐channeling” approach in which conductive channels are identified by means of high‐density voltage mapping or electrogram analysis. Radiofrequency applications at the entrance to these conductive channels can block conduction inside (a part of) the scar without extensive ablation.^15^ (Figure 1E).

Pace mapping consists in pacing from areas of abnormal electrogram (EGMs) in and around the scar, in an attempt to match the clinical VT morphology, and can help to approximate the anatomic location of VT.^9^ Pacing from within the scar can also identify slow‐conduction channels, which are marked by a prolonged stimulus to QRS interval (S‐QRS),^16^ while pacing at the VT exit site will yield a “matched” QRS with a short S‐QRS. Recently, it has been recognized that an abrupt transition between a paced‐QRS that matches the clinical VT (exit site) and a nonmatched paced‐QRS (entrance site) can identify an isthmus.^17^

Lately, techniques involving electrical isolation of the scar (scar isolation and core isolation, Figure 1F) have been developed.^9^ Tilz et al.^18^ hypothesized that, in selected post‐myocardial infarction (MI) patients with a circumscribed scar, encircling the arrhythmogenic area would be feasible and cause electrical dissociation of the isolated area from the remainder of the left ventricle, while Tzou et al.^19^ proposed the “core isolation” approach. This is a stepwise approach that starts identifying critical or core VT circuit elements based on careful electrophysiological characterization and ablating these areas circumferentially with the goal of achieving electric isolation.

Scar homogenization seems to have a higher success rate in terms of both acute success (inducibility at the end of the procedure) and VT free survival compared with the other ablation strategies (Table 1). However, the fact that more ablation is more effective probably means that selective mapping and ablation methods have a low specificity.

**Table 1:** 

	Publication year	No of patients	Ischemic cardiomyopathy	No ischemic cardiomyopathy	Mapping technique	Strategy for ablation	Acute successful (no VT inducibility)	Death	Follow‐up duration	VT recurrence
Marchlinski et al.^4^, ^25^	2000	16	9	7	Carto System	LINEAR ABLATION	8 (50%)	0%	36 months	25%
Vergara et al.^8^	2012	64	41	23	Carto System‐Navx Ensite Velocity	LATE POTENTIAL (50pts)	37 (74%)	0%	13 months	20%^a^
Jais et al.^7^	2012	70	56	14	Carto System	LAVA (Local Abnormal Ventricular Activities) 67pts	49 (73%)	0%	22 months	46%^a^
Di Biase et al.^6^	2012	92	92	0	Carto System	HOMOGENIZATION OF SCAR (43 pts.)	43 (100%)	0%	22 months	19%
Berruezo et al.^15^	2015	101	75	26	Carto System‐Navx Ensite Velocity	CONDUCTING CHANNEL	55^b^ (55%) 79^c^ (78%)	0%	21 months	Not Assessable^c^
Tzou et al.^19^	2015	44	32	12	Carto System	CORE ISOLATION	32^b^ (72%) 37^c^ (84%)	0%	18 months	Not Assessable^c^

Abbreviation: VT, ventricular tachycardia.

a Referred to total populations.

b Patients ablated according with Ablative Study Strategy.

c Patients who underwent Ablative Study Strategy + Other Ablative strategies if VT was again inducible.

The above‐described strategies of substrate ablation can be implemented not only in the endocardium, but also in the epicardium when an epicardial approach is adopted. In patients with ischemic cardiomyopathy, epicardial ablation is most commonly undertaken after failed endocardial ablation, although in some studies greater freedom from recurrence has been reported when a combined endocardial‐epicardial approach is performed from the beginning.^20^ However, in common practice, not uniform approach is utilized in VT ablation and at least 2 to 3 methods should be combined to identify critical circuit areas. For example, in the same patient, activation, and entrainment mapping can be used for inducible/tolerated VT, while 1 or 2 of the substrate mapping strategies is utilized for not inducible/not tolerated VT. Again, voltage mapping and LPs mapping an ablation are commonly used during the same procedure in patients with not tolerated VTs.

## 3 THE LIMITATIONS OF SUBSTRATE MAPPING IN PATIENTS WITH ISCHEMIC VT

Ineffectiveness of VT ablation is probably related both to the inadequacy of current mapping strategies and to the limited depth and extent of myocardial injury created by radio frequency ablation, especially in patients with intramural substrate when significant portions of the re‐entry circuit are deep to the endocardium, beyond the limits of catheter ablation using standard irrigated catheters. In the situation of arrhythmogenic substrate remote from the site of radio frequency application, adjunctive ablation techniques can be helpful, including: ablation at high powers for a prolonged duration, ablation from multiple sites surrounding the arrhythmogenic site using simultaneous unipolar or bipolar configuration, the use of an irrigated needle tip catheter in an effort to reach the deep intramural substrate, and the use of noninvasive stereotactic radio‐ablation which consists of irradiation of the arrhythmogenic area in a manner similar to tumor radiotherapy.^21^, ^22^, ^23^, ^24^

In summary, over the years, many efforts to facilitate catheter ablation of post–myocardial infarction VT have been made. The substrate‐based VT ablation techniques, which require specific technology to characterize epicardial or endocardial substrates, have played a leading role in prompting the industry to improve catheter and mapping technologies. Nevertheless, substrate mapping may fail to accurately delineate the extent of diseased myocardium and the critical VT isthmus where ablation should be performed.

Indeed, all the substrate mapping procedures described are based on one or more of the following strategies, which present some major challenges:

1. definition of low‐voltage areas (scar, dense scar, transitional border zone) by unipolar and bipolar voltage mapping.
2. recording of abnormal electrograms (LAVA and/or LPs).
3. localization in sinus rhythm of channels defined by fixed anatomical barriers.

## 4 DEFINITION OF LOW‐VOLTAGE AREAS

Marchlinski^25^ defined the normal and abnormal endocardial voltage values during sinus rhythm identifying the dense scar area, the border zone and the normal myocardium in relation to the presence of voltage amplitude<0.5 mV, between 0.5 and 1.5 an >1.5 mV, respectively. It was also shown that epicardial bipolar voltage should be considered abnormal when a signal amplitude<1.0 mV is recorded and abnormal electrogram characteristic (wide, split, LPs) are present, while voltage amplitudes of 0.5 to 1.0 mV define the border zone and area with bipolar signal amplitude<0.5 mV is defined as scar.^26^ Moreover, it was established that unipolar endocardial mapping can identify the presence and location of bipolar low voltage regions localized in the epicardium or in intramural sites in patients with dilated cardiomyopathy who do not demonstrated endocardial bipolar abnormalities. The normal signal amplitude for left ventricular endocardial unipolar electrogram was >8.7 mV.^26^

**Figure 3:**
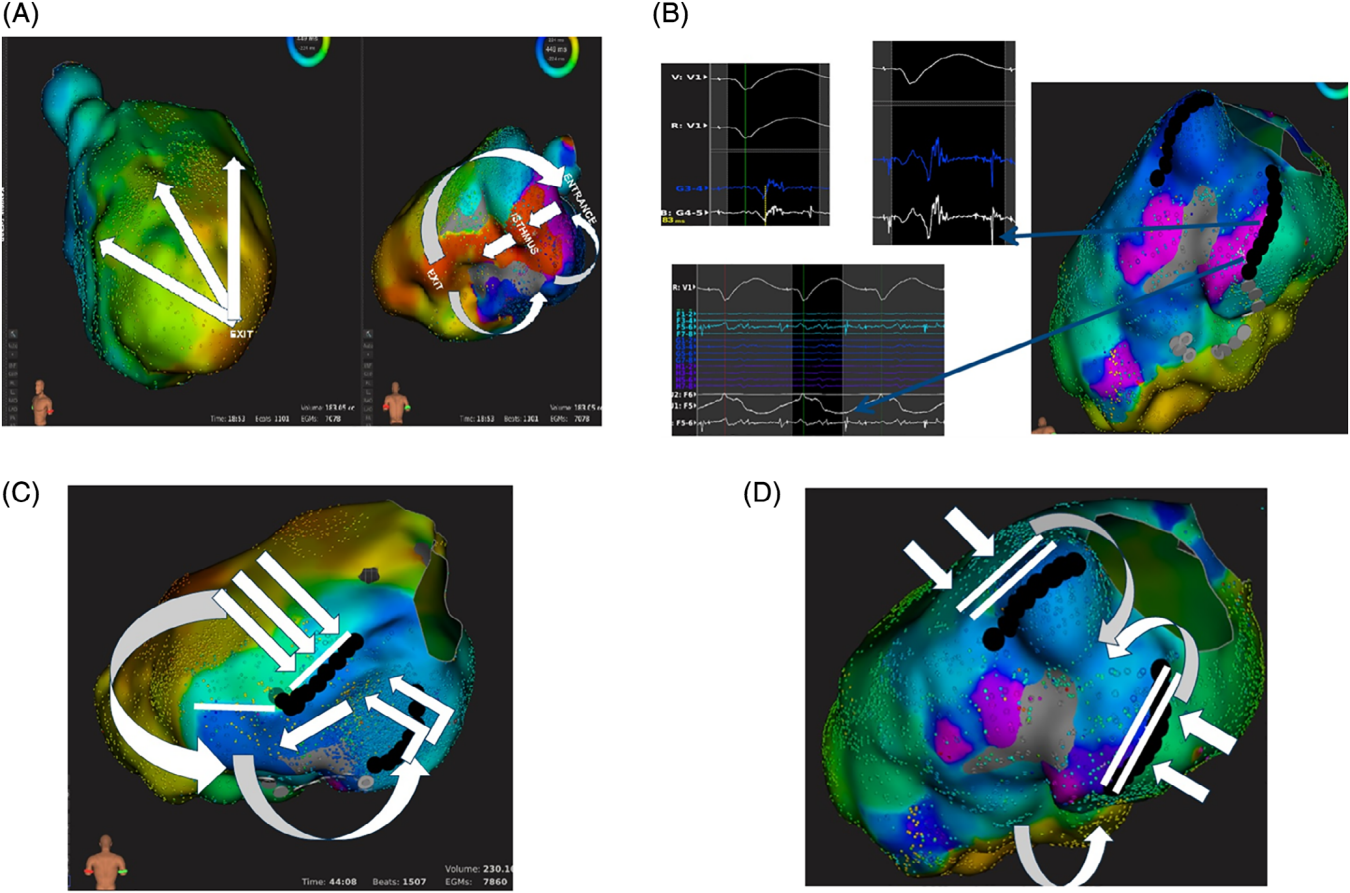
Evidence of a functional circuit in a patient with ventricular tachycardia (VT). A, The activation map of the VT is shown with the full circuit from the entrance to the exit sites, passing through the VT isthmus, which is delimited by two lateral boundaries of conduction block. B, An activation map during sinus rhythm, with the superimposed lateral boundaries present during VT. It is evident that, during sinus rhythm, only the lateral barrier of conduction block on the left of the map is present, while the lateral boundary on the right is completely absent. C, The activation map during right ventricular pacing at a cycle length approximating the VT cycle length shows the presence of the two lateral barriers present during VT. These data demonstrate that the left lateral boundary is anatomical and fixed, while the right lateral boundary is functional in nature. D, Double potentials, recorded during VT and during pacing from the right ventricular apex, along the functional barrier

It is common practice to identify scar tissue and fibrosis as low‐voltage areas, on the assumption that bipolar voltage is a reflection of the underlying tissue. However, the voltage amplitude recorded by the mapping catheter does not always correspond with areas of true scarring and vice versa. Indeed, the recorded electrogram voltage is related to many factors. First of all, the direction of the activation wavefront influences the bipolar and unipolar voltage amplitude. In this regard, significant differences have been found in the bipolar and unipolar voltage of scar tissue, according to the ventricular activation wavefront (Figure 3). In one study, the median bipolar and unipolar scar area was seen to vary by 22% and 14%, respectively, as the activation wavefront varied.^27^ As critical sites for VT may be localized in regions that are normal during one wavefront, mapping an alternate activation wavefront is therefore useful in order to increase the sensitivity in detecting the arrhythmogenic substrate. The angle of incidence, that is, the orientation of the catheter relative to the tissue, determines the location of the electrode relative to the tissue, and thus the amplitude of the bipolar recordings.^28^ Electrode size and interelectrode spacing also affect the amplitude of the bipolar electrogram, as well as conduction velocity.^28^ Finally, the tissue contact and the filtering used affect the amplitude of the electrogram.^28^ These findings suggest that the electrogram amplitude may not truly reflect the histological status of the directly recorded myocardium (bipolar electrograms) and even of the underlying myocardium (unipolar recordings). These observations have been confirmed by studies comparing electroanatomic mapping and magnetic resonance imaging, with or without delayed contrast enhancement,^29^ which have revealed the limited accuracy of electroanatomic mapping in delineating precisely scar extension.

## 5 RECORDING ABNORMAL ELECTROGRAMS

In many substrate mapping strategies, abnormal electrograms are the targets of mapping and ablation. Some studies have regarded all abnormal electrograms (LAVA) as the ablation target,^7^ while others have considered only LPs.^9^

The morphology and time of onset of electrograms are related to the direction of the propagating impulse.^30^ (Figure 2).

Thus, hidden abnormal/LPs may not be considered for ablation if only the abnormal/LPs present during one specific rhythm are considered. Sacher et al.^13^ showed that pacing maneuvers are sometimes required in order to distinguish a local abnormal electrogram from the far‐field ventricular signal. Brunckhorst et al.^31^ assessed the effect of a change in activation sequence on the bipolar electrogram in infarct regions. They found that multicomponent electrograms disappeared and appeared on changing the activation sequence, and that abnormal electrograms were often present during only one of the two activation sequences. Finally, also the use of ventricular extrastimuli may be useful in unmasking abnormal electrograms hidden within the far field signal.^32^

## 6 LOCALIZATION IN SINUS RHYTHM OF CHANNELS DEFINED BY FIXED ANATOMICAL BARRIERS

Most of the substrate mapping and ablation strategies are based on the assumption that re‐entrant circuits and channels are anatomically defined, fixed and present in sinus rhythm although many have demonstrated that re‐entrant circuits in postmyocardial infarction VT are, at least in part, functional. Moreover, we do not yet have any certain surrogate that can identify in sinus rhythm the site of a potential barrier or channel during VT. In fact, the characteristics of a functional barrier is that block or pseudo‐block are present during VT but not in sinus rhythm. Several studies, both in animals and in humans, have demonstrated the presence of functional circuits sustaining postinfarction VT. More recently, in a swine model of infarct‐related re‐entrant VT, Anter et al.^33^ demonstrated that the arrhythmogenic substrate displayed zones of slow conduction due to nonuniform anisotropy, resulting in fixed and/or functional regions of conduction block. Segal et al.^34^ investigated the mechanism underlying human postinfarct VT initiation by using noncontact mapping. They found that initiation of sustained monomorphic VT required the development of unidirectional block and the formation of lines of functional block creating the borders for the diastolic pathway in areas of slow conduction. In our Lab (unpublished data), we have observed the presence of lines of conduction block forming the lateral barriers of the VT circuit during VT and ventricular pacing, but not during sinus rhythm (functional block) (Figure 3).

Several mechanisms have been proposed to explain the development of functional block, all of which can probably contribute to the origin of functional re‐entry in the same or different situations. The first mechanism involves a defect in the number and function of gap junctions, leading to the conversion of conduction properties from uniform to nonuniform anisotropy^35^ (anisotropic re‐entry). The second mechanism involves regional differences in ionic currents in cells of the border zone, leading to alteration of the depolarization and repolarization phases of the action potential^36^ and then to the inhomogeneous changes in refractory periods and refractoriness dispersion that form an adequate substrate for unidirectional block and the initiation of functional re‐entry. A third mechanism proposed to explain functional conduction block or pseudo‐block leading to the onset of the lateral barriers of a functional re‐entry is based on impedance or source‐sink mismatch.^37^ In areas like the infarct border zone, block can occur at sites of discontinuity in lateral boundaries where there is a sharp transition from thin‐to‐thick tissue (from isthmus to lateral boundaries). This occurs because the available current is insufficient to activate the greater volume of tissue in the thin‐to‐thick direction sometimes already in normal conditions but more frequently after a premature beat with a short coupling interval or during a short cycle length rhythm such as that present during VT.^38^ Indeed, the wavefront curvature becomes convex as it travels from a lesser to a greater volume, and when the curvature becomes critically convex, functional conduction block will occur, owing to the difficulty in delivering a sufficient electrical charge to the larger volume of tissue distal to the activating wavefront (source‐sink mismatch) (Figure S2).^38^ If a difference in thickness is present, but not crucial, a critical convex curvature will not be attained, conduction block will not occur, and only slow conduction through the discontinuities of lateral boundaries (pseudo‐block) will ensue. In the light of the above considerations, substrate mapping and ablation have important limitations, which should lead to substantial changes in ablation procedures and fuel the search for new ablation targets, bearing in mind that:

1. as several factors affect the amplitude of both unipolar and bipolar recordings, there is no reliable method of achieving accurate scar delimitation.
2. LAVA/LP recordings are influenced by wavefront activation, pacing rate, programmed stimulation, site of recording (septal vs lateral sites); consequently, there is no reliable method of detecting all the potentials inside the scar and at the border zone that may be the hallmarks of anatomic/functional channels.
3. functional barriers and channels have an important role during VT, but, at the moment, there is no reliable method of defining in sinus rhythm the sites of potential barriers and channels during VT.

How can we curtail the limitations of substrate mapping described in the previous paragraphs?

Regarding the first two points, it is evident that the use of high‐density mapping by means of small electrodes with short center‐to‐center inter‐electrode spacing may help to reduce some of the misinterpretations of conventional substrate mapping with standard ablation catheters with a 3.5 or 4 mm tip and a larger inter‐electrode distance. Although bipolar voltage amplitude in the healthy ventricle is similar between linear (standard) and multielectrode high‐density mapping catheters, mapping resolution within areas of low voltage and scar is enhanced with multielectrode catheters that identify areas of preserved myocardial bundles (channels) otherwise considered dense scar by standard linear catheters.^39^

**Figure 4:**
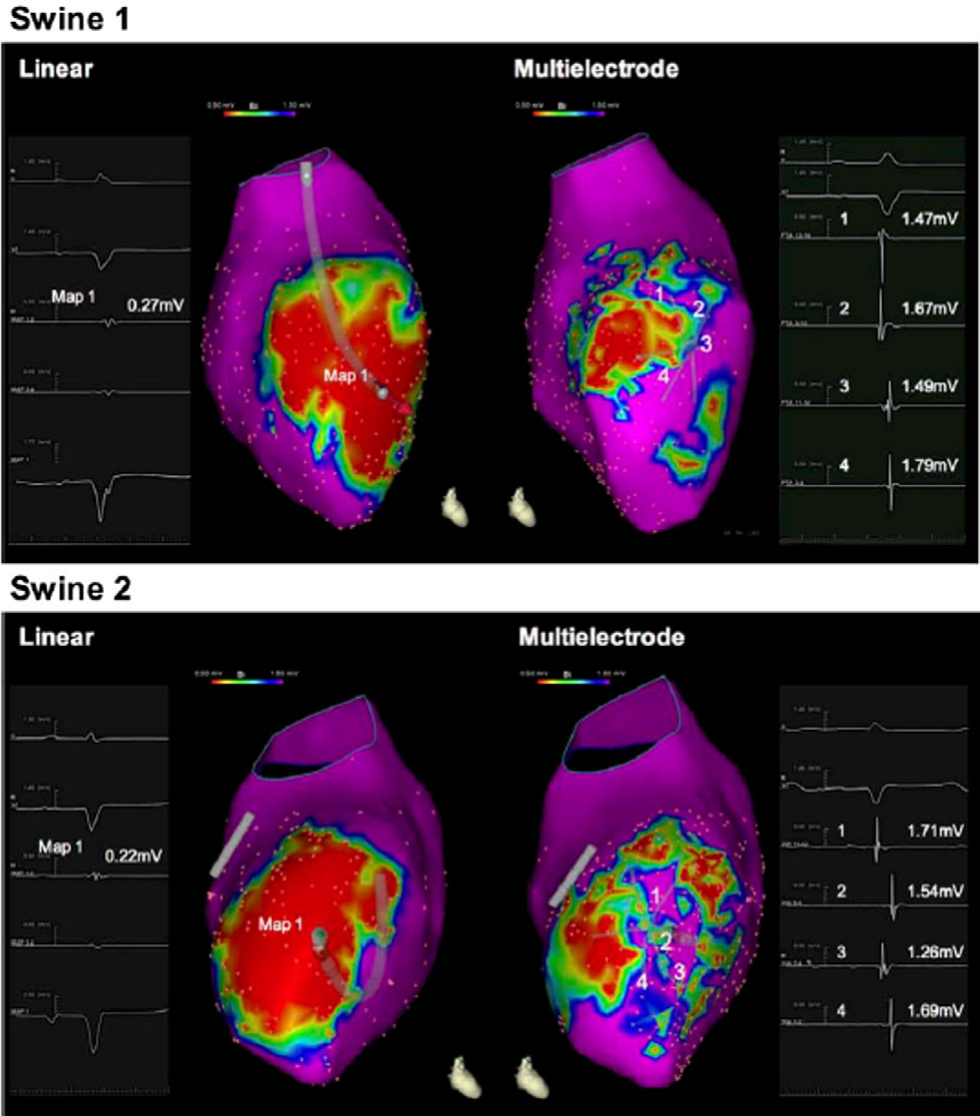
Electroanatomic clinical maps (EAM) of the left ventricle in a postinfarction heart. The EAM made with a standard ablation catheter is shown on the left, whereas the EAM made with the multi‐electrode catheter (high‐resolution mapping) is shown on the right. Mapping with a standard ablation catheter demonstrated the presence of a homogenous and confluent low‐voltage area, while high‐resolution mapping revealed channels of normal bipolar voltage amplitude in the same area (see Reference 47)

Indeed, the use of high‐density mapping with small electrodes and short center‐to‐center inter‐electrode spacing enables surviving myocardial fibers, that show a higher voltage signal, to be identified inside heterogeneous low‐voltage areas; this approach differs from low‐density mapping by means of standard ablation catheters, which records activity from a large area and is associated with high interpolation between points. High‐density mapping is expected to produce a more reliable ventricular map with a better resolution, on which the scar, border zone and viable myocardium outside and inside the scar are more precisely delineated (Figure 4). In addition, mapping with multi‐electrode catheters yields more data points and better variability in the angle of incidence and in the relationship to the vector of propagation, thereby reducing the individual confounding effects of bipolar voltage amplitude measurements. Finally a recent paper^40^ has shown that endocardial catheter measurement of the electrical impedance can identify infarct scar regions and, in contrast to voltage mapping, the impedance data is not affected by changes in cardiac activation sequence.

Furthermore, the recognition of LP and/or LAVA may be improved by the use of high‐resolution mapping performed with multipolar catheters equipped with small electrodes and with short inter‐electrode spacing, as this approach facilitates visualization of the near‐field potential inside the far‐field potential. Moreover, as high‐resolution mapping enables rapid and precise mapping, activation and substrate maps can quickly be created both during sinus rhythm and during ventricular paced rhythms (from the right or left ventricle), thereby unmasking abnormal electrograms that are present during only one of the two different ventricular activation sequences.^41^ The same arguments are valid also when identification of local abnormal electograms hidden within the far field signal has to be achieved by mean of a double extrastimuli delivering.^32^

Regarding the third point, it could be of paramount importance to identify, in sinus or paced rhythms, the morphological electrogram features, the EPS characteristics and the specific structural anatomic and geometric hallmarks of a site that will become the functional barrier or the isthmus of a functional re‐entrant circuit. Recent studies suggest that such sites, and therefore possible new targets for ablation, could be:

1. sites where recorded electrograms show lateness, low Shannon entropy and low SD in the activation time of their components.^42^
2. sites that show sudden thin‐to‐thick transition on high‐resolution Magnetic Resonance Imaging (MRI).^27^
3. sites showing long Effective refractory period (ERP), long total recovery time, a wide refractoriness dispersion^35^, ^36^ or a high vulnerability to reentry (see below).

A recent paper by Nayyar et al.^42^ reported that sinus rhythm electrograms residing within the location where the isthmus forms during VT are often characterized by multiple deflections, late mean activation time of the deflections, greater disparity in the time of deflections and lesser value of Shannon entropy (narrow voltage distribution). Ablating the regions with these electrogram criteria enabled the authors to eliminate many of the clinical VT that were inducible in each patient. This new mapping method can facilitate recognition of functional VT channels (isthmus and/or lateral barriers) and allow substrate mapping and ablation also of functional re‐entrant VT circuits.

Another possible method usable during sinus rhythm in order to localize sites where a VT isthmus will develop requires the use of cardiac MRI, which unfortunately at the moment is not available for human use. Moreover, even the sites of functional barriers present only during VT could be identified in sinus rhythm by cardiac MRI. In fact, functional block during VT occurs at the lateral isthmus boundaries, where there is a sharp change from the thinnest to the thickest infarct border zone.^42^ As previously discussed, this sharp thin‐to‐thick transition can also result in critical wavefront curvature and impedance or source‐sink mismatch, causing functional conduction block.^37^ Therefore, if we acquire high‐resolution cardiac MRI images (at the moment not available for human use) regions with significant thinnest‐to‐thickest geometry could be detected and located finally. In a recent animal study a negative relationship between conduction velocity and wall thickness gradient was observed, where conduction velocity decreases 0.05 m/s per unit increase in wall thickness gradient. Region of steeper wall thickness gradient represent the infarct border zone where the wave‐front curvature changes resulting in further conduction velocity slowing or block.^43^ Regions with this specific structural configuration correspond to segments with the specific electrogram characteristics described by Nayyer et al.^42^ Using this method would allow most of the procedural time to be spent ablating arrhythmogenic areas and obviate the need to induce the VT or perform extensive scar mapping.

Another mechanism invoked for the initiation of a functional re‐entry is dispersion of refractoriness and of total recovery time^44^ as shown in canine infarcts models.^36^ Therefore, sites with prolonged refractoriness and/or prolongation of total recovery time could be another ablation target during sinus rhythm.

Finally, recent studies are trying to go beyond voltage combining substrate structures analysis with tissue function properties for identifying the complex arrhythmogenic substrate. The new concept of the reentry‐vulnerable zone has been proposed by Anter et al.^45^ It refers to the existence of a limited part of the scar and peri scar zone where all the reentrant circuit develops, identified by sites showing a steep activation gradient and a high curvature, and represents a new strategy of substrate VT mapping and ablation. Also the reentry vulnerability index to pick out region of reentry represents another possible method to predict localized regions of high susceptibility to reentry based on the activation‐repolarization time metric that is calculated from intervals between local repolarization and activation times.^46^

## 7 CONCLUSIONS

Despite the technical improvements made in recent years, the overall long‐term success rate of VT ablation in patients with ischemic cardiomyopathy remains disappointing. This unsatisfactory situation has persisted even though several approaches to VT substrate ablation allow mapping and ablation of noninducible/nontolerated arrhythmias. The current substrate mapping methods present some shortcomings regarding the accurate definition of the true scar, the modality of detection in sinus rhythm of abnormal electrograms that identify sites of critical channels during VT and the possibility to determine the boundaries of functional re‐entrant circuits during sinus or paced rhythms. The last point seems to be of great importance, since animal and human studies have shown that re‐entrant circuits in postmyocardial infarction VT are, at least in part, functional and not anatomically defined. Recent studies have assessed the electrogram features, EPS characteristics and structural anatomic/geometric hallmarks of sites that will become the channels (isthmus and/or lateral barriers) of the re‐entrant circuit during VT. It has been speculated that alternative approaches to VT ablation based on searching for these specific electrogram or EPS hallmarks or for specific geometric/anatomic peculiarities detected by cardiac MRI will prove effective. The validity of these new possible approaches to VT ablation requires future studies in order to compare the effectiveness of the proposed strategies with the currently used methods of catheter ablation in ischemic cardiomyopathy patients with VT.

## CONFLICT OF INTEREST

Maurizio Malacrida is an employee of Boston Scientific; no other conflict of interest exist.

## Supporting information


**Figure S1.** Examples of local abnormal ventricular activity (LAVA) (A) and late potential (LP) (B).

**Figure S2.** In areas such as the infarct border zone, block or very slow conduction can occur at sites of discontinuity in lateral boundaries, where there is a sharp transition from thin‐to‐tick tissue (from isthmus to lateral boundaries). This occurs because the available current is insufficient to activate the greater volume of tissue in the thin‐to‐thick direction. Indeed, during electrical impulse propagation, the wavefront curvature becomes convex as it travels from a lesser to a greater volume; when the curvature becomes critically convex, because of a sudden large change from lesser to greater volume in the direction of travel, functional conduction block (A) or very slow conduction with variable propagation across the lateral boundaries (B) will occur. The cause lies in the difficulty in delivering sufficient electrical charge to the larger volume of tissue distal to the activating wavefront (source‐sink mismatch). *Source*: Reference 38 with permission).
