# Supplementary figures and images for: Mapping of ventricular tachycardia in patients with ischemic cardiomyopathy: Current approaches and future perspectives
Source: Clin Cardiol. 2019 Aug 14;42(10):1041–50. doi: 10.1002/clc.23245 (PMC6788471; doi:10.1002/clc.23245)

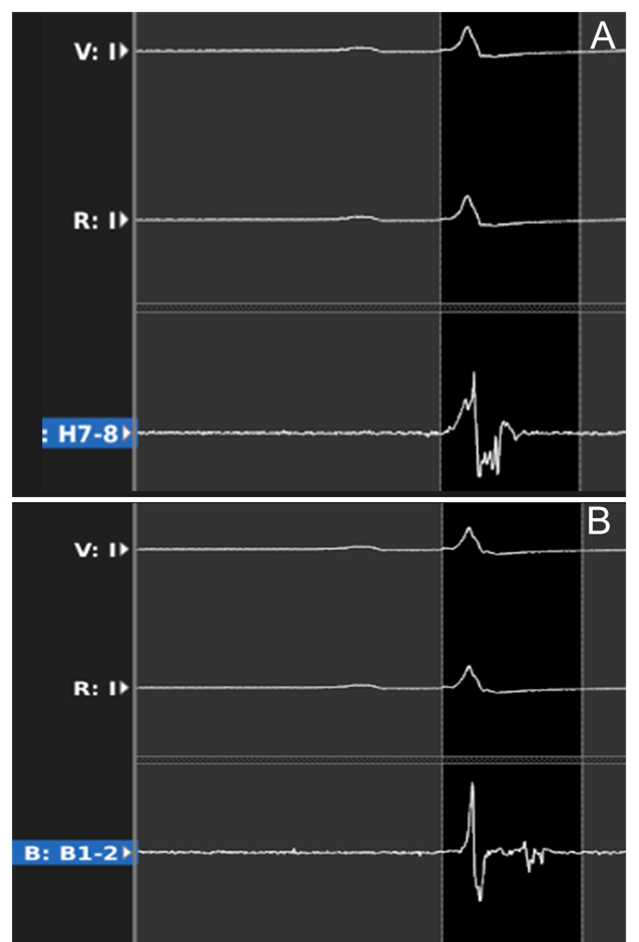

Supplement: Supplementary file 1 — Figure S1. Examples of local abnormal ventricular activity (LAVA) (A) and late potential (LP) (B). [file CLC-42-1041-s001.tif]

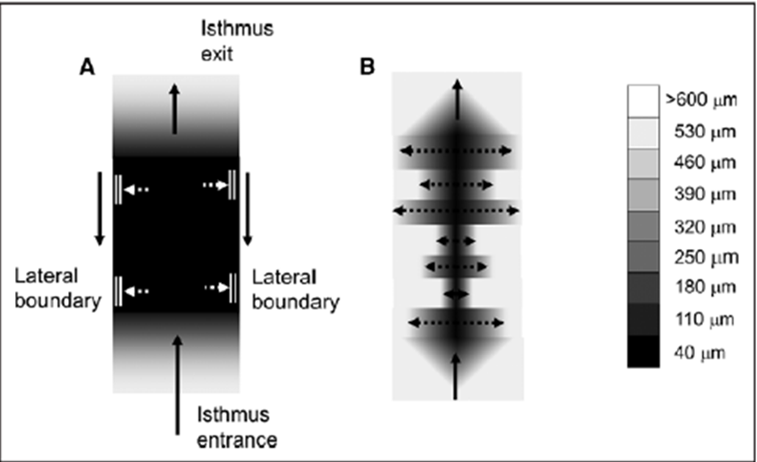

Supplement: Supplementary file 2 — Figure S2. In areas such as the infarct border zone, block or very slow conduction can occur at sites of discontinuity in lateral boundaries, where there is a sharp transition from thin‐to‐tick tissue (from isthmus to lateral boundaries). This occurs because the available current is insufficient to activate the greater volume of tissue in the thin‐to‐thick direction. Indeed, during electrical impulse propagation, the wavefront curvature becomes convex as it travels from a lesser to a greater volume; when the curvature becomes critically convex, because of a sudden large change from lesser to greater volume in the direction of travel, functional conduction block (A) or very slow conduction with variable propagation across the lateral boundaries (B) will occur. The cause lies in the difficulty in delivering sufficient electrical charge to the larger volume of tissue distal to the activating wavefront (source‐sink mismatch). Source: Reference 38 with permission). [file CLC-42-1041-s002.tif]
